# Some Physicochemical Remarks on Spontaneous Emulsification of Vitreal Tamponades

**DOI:** 10.1155/2014/243056

**Published:** 2014-07-15

**Authors:** Ciro Costagliola, Francesco Semeraro, Roberto dell'Omo, Lucio Zeppa, Gennaro Bufalo, Michele Cardone, Mario Romano, Luigi Ambrosone

**Affiliations:** ^1^Department of Medicine and Health Sciences, University of Molise, via De Sanctis, 86100 Campobasso, Italy; ^2^Department of Ophthalmology, University of Brescia, Piazzale Spedali, 25123 Brescia, Italy; ^3^Department of Ophthalmology, G. Moscati Hospital, Contrada Amoretta, 83100 Avellino, Italy; ^4^Department of Naples, INAIL (ex ISPESL) Settore Ricerca, Certificazione Verifica, 80143 Naples, Italy; ^5^Department of Ophthalmology, Federico II University, Via Pansini, 80131 Naples, Italy; ^6^Department of Bioscience and Territory, University of Molise, Contrada Conte Lappone, Pesche, 86090 Isernia, Italy

## Abstract

The importance of gravitational instability in determining the emulsification of vitreal tamponades is discussed. Theoretical results and numerical simulations indicate that the spontaneous formation of water-silicon oil is a rare event and that the very low concentration of surface active agents cannot justify the systematic formation of emulsions. The gravitational instabilities seem to play the main role. Our theoretical results seem in agreement with the experimental evidences; furthermore they indicate a future research line for the improvement of endotamponades. Indeed, the use of biodegradable antifoam may avoid the formation of bubbles and delay the formation of emulsions.

## 1. Introduction

Intraocular tamponade agents have been used by vitreoretinal surgeons for a long time to repair retinal detachment, a potentially blinding condition with an incidence reported to be between 6.3 and 17.9 per 100000 [1, 2]. Traditional tamponade agents include gases and silicone oils that, owing to their lower specific gravity, provide good support for breaks or holes located in the upper retina [3]. For retinal holes in the lower retina, tamponades which are heavier than water offer a more logical approach. Among them, perfluorocarbon (PFCL) liquids have an important role for intraoperative manipulation of retina but may induce retinal degeneration after long-term use [4]. Similarly, semifluorinated alkanes such as perfluorohexyloctane (F6H8) are associated with early and extensive emulsification and are therefore not used as long-standing tamponade agents. Conversely, heavy silicone oils, also called fluorosilicones, obtained by mixing silicone oil with semifluorinated alkanes, are well tolerated and offer a satisfactory support for the inferior retina.

Both silicones and fluorosilicones emulsify after incorporation into the eye [5]. However, from the thermodynamic point of view, spontaneous emulsification can only occur under specific conditions depending on the chemical composition of phases and the presence of surface active agents (*surfactants*) [6–8]. What is the mechanism of formation of emulsions in the vitreous cavity? Which are the surfactants in the eye? Herein we focus mainly on the chemical-physical properties of the tamponades to address these questions.

## 2. Chemical Structure and Surface Properties

The surface properties of silicones (or more exactly organosiloxane polymers), closely related to their unique chemistry, are responsible for many of their applications. The polydimethylsiloxanes (PDMS) are the most common and possess the most interesting surface properties [9, 10].

The general structure of these colorless liquids insoluble in water is (CH_3_)_3_SiO[(CH_3_)_2_SiO]_*n*_Si(CH_3_)_3_, with *n* approximately 0 to 2500. From comparison with other polymers, one deduces that the surface activity of PDMS approximates that of a relatively close-packed array of methyl groups. These properties are also characteristic of organic polymers, which are however handicapped by thermal and oxidative instability [11]. The fluorocarbons, characterized by lower surface energy than PDMS, have a high interfacial tension in both aqueous and organic solvent systems. In both systems it has been observed that methylsilicones were less emulsified than fluorosilicones of same viscosity, suggesting that the smaller density difference between silicones and intraocular fluid makes intermixing with water more difficult as compared with fluorosilicones [12].

## 3. Emulsification Formation

Generally an emulsion is defined as two immiscible liquids wherein droplets of one phase (*the dispersed phase*) are spread in a* continuum* of another phase (*the continuous phase*) [13–15]. When silicone oil is introduced in the eye, two basic forms of emulsions are possible. The first is a silicone oil-in-water (o/w) emulsion, in which silicone droplets are dispersed within a continuum of water. The second is a water-in-silicone emulsion (w/o), where water droplets are dispersed in a continuum of silicone. Davis and Rideal suggested that both types of emulsions are formed during the emulsification process, but only the one with the lower coalescence rate survives [16]. Indeed, if the initial concentration of drops is the same for both types of emulsions the coalescence rate *v*_1_ for type o/w, *v*_2_ for type w/o, and their corresponding interfacial film lifetimes, *τ*_1_ and *τ*_2_, are inversely proportional:

(1) $$\begin{array}{c} \frac{v_{1}}{v_{2}}=\frac{\tau_{2}}{\tau_{1}}. \end{array}$$

By evaluating *τ*_1_ and *τ*_2_ using the lubrication's theory one proves that the emulsion, where the surfactant is soluble in the continuous phase, will coalesce much more slowly and thus it will survive [16]. In the vitreous cavity silicone oil is in the presence of vitreous liquid; that is to say, a biphasic system is created. The water promotes the formation of w/o emulsions. On the other hand, however, in the vitreous cavity there are also compounds such as phosphatidylcholine and proteins, which form o/w emulsions. The rate of formation of emulsions is proportional to the concentrations of surfactants. Indeed, it was observed that high level of cholesterol in the eye is associated to high rate of emulsions of the type w/o emulsions [17].

## 4. Free Energy of Emulsion Formation

In order to allow emulsification to occur, a work to increase the interfacial area between two liquids has to be done. Such a process is accompanied by change in free energy of formation:

(2) $$\begin{array}{c} \Delta G^{\text{form}}=\gamma \Delta A-T\Delta S^{\text{conf}}, \end{array}$$

where *γ* is the mean interfacial tension, *γ*Δ*A* is the work done to increase the interfacial area of Δ*A*, *T* is the absolute temperature, and Δ*S* ^conf^ is the entropy change due to the different system configuration. The emulsification process is spontaneous if Δ*G* ^form^ < 0. This can be achieved only if the work done on the system (in absolute terms) is less than the entropic contribute. This result can be achieved in two ways: by increasing Δ*A* or by reducing *γ*. The first is generally obtained by blowing mechanical energy in the system, while the second by means of surfactants which reduce the surface tension. Since mechanical energy blow in the eye is a nonsense, only the second way seems to be possible. Thus the presence of a mixture of surfactants could generate an ultralow (or transiently negative) interfacial tension so that the work, to create the new surface, becomes comparable or even lower than the configurational entropy. In this case the variation of free energy of formation would be zero or negative, and the process would appear to be spontaneous. A rough estimate of the chance that a low interfacial tension in the eye is sufficient to cause spontaneous emulsification can be obtained by calculating the two different terms of (2). Indeed, according to Tadros and Vincent [18], the configurational entropy can be estimated by

(3) $$\begin{array}{c} \Delta S^{\text{conf}}=-nk_{B}\left[\ln\phi -\frac{1-\phi }{\phi }\ln\left(1-\phi \right)\right], \end{array}$$

where *k*_*B*_ is the Boltzmann constant, *n* is the droplets number, and *ϕ* is the volume fraction of the dispersed phase. On the other hand, when *n* droplets of radius *R* are formed, the surface increase is Δ*A* = *n*4*πR* ^2^; thus (2) and (3) allow determining the emulsion spontaneity as a function of volume fraction of the dispersed phase, when the interfacial tension *γ* and the average radius *R* are known. For in water-emulsions we assume an average radius of 5 *μ*m and calculated the free energy change per droplet as a function of the volume fraction and for various values of interfacial tension.

**Figure 1:**
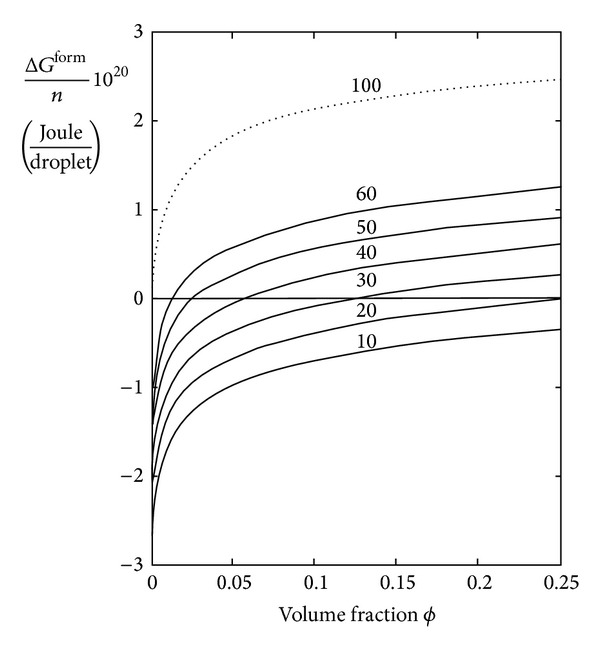
Change in free energy for the formation of *n* water droplets in a heterogeneous system water-silicone oil at 37°C, as a function of volume fraction of droplets of radius *R* = 5 *μ*m.

From Figure 1 it is evident that droplets formation with a mean radius of 5 *μ*m is spontaneous only for interfacial tensions very low, of the order of 10^−12^ Nm^−1^. In fact, a value of only 10^−10^ Nm^−1^ suffices to make the free energy positive. Such very low values are not achievable, ruling out the possibility that an ultralow interfacial tension is responsible for spontaneous emulsification of liquid tamponades. It is important to note that such a conclusion is valid for any type of liquid tamponade independently of its density or viscosity.

## 5. Surfactant Role

Generally emulsions formed in the eye have large size; to understand this aspect from physical-chemical point of view, we assume that the disperse phase is a large drop of prolate spheroidal shape [19–21]. The equilibrium geometry of the two phases is dictated by the pressures in the two phases which are related by Laplace equation:

(4) $$\begin{array}{c} \Delta p=\gamma \left(H_{1}+H_{2}\right), \end{array}$$

where Δ*p* is the difference in pressure between inside and outside of the drop and *H*_1_ and *H*_2_ are the principal curvatures [22, 23]. In the case of a spherical drop *H*_1_ = *H*_2_ = *H*. A large drop can be broken up into many small drops if it is strongly deformed with great values of Δ*p*. But, as one can see from (4), a spherical drop has only one curvature while a prolate spheroid (or even a generic form) has two curvatures so that the stress necessary to deform a small drop is higher than that required to deform a large one. In addition, since the stress is not transmitted directly to the drops but to the liquid surrounding the drops, the energy required to produce the deformation is even higher. However, the surfactant ability to lower the interfacial tension depends on its concentration at the water-tamponade interface as well as on the energetic interactions of the surfactants with the surrounding phases. Although the amount of surfactants present in the eye is not sufficient to disperse the water phase into small droplets, the presence of such substances is of fundamental importance for the stability of the system. Depending on their individual rate of interfacial adsorption as well as their amphiphilicity, different surfactants lower the interfacial tension to a different extent during emulsion formation, thereby affecting the final size distribution of the emulsion droplets. There are obvious differences between the surface properties of a low-molecular-weight surfactant and biomolecules. In small molecules the amphiphilic character of the molecular structure is easily delineated. This simple structure allows the molecule to adopt a low-energy conformation at the interface and its small size leads to high packing density. The structure of a protein emulsifier is obviously more complicated than that of a low-molecular-weight surfactant and can less readily be described by the idealized head-tail model. Hydrophobic groups, consisting of nonpolar amino acids, are distributed throughout the protein molecule, and their ability to access a nonpolar phase at an interface can require complex rearrangements of the native protein. In addition, emulsion formation is directly affected by emulsifier concentration because this concentration determines the surface excess of surfactant and hence the degree to which the interfacial tension is lowered. Another important role of the surfactant is its effect on the interfacial dilatational modulus [24]. During emulsification there is an increase in the interfacial area *A* and this causes a reduction in the surface excess. The equilibrium is restored by adsorption of surfactant from bulk, but this takes variable times, depending on surfactant concentration. The presence of more than one surfactant molecule at the interface tends to increase the interfacial dilatational modulus. Surfactants may vary in surface activity and this regulates their distribution at the interface. Indeed, surfactants with the lowest *γ* tend to predominate at the interface, but if present at low concentrations, it may take long time to reach the lowest value. In the vitreous the protein concentration is very low, so that very long times are expected in order to start the process.

## 6. Hydrodynamic Aspects

**Figure 2:**
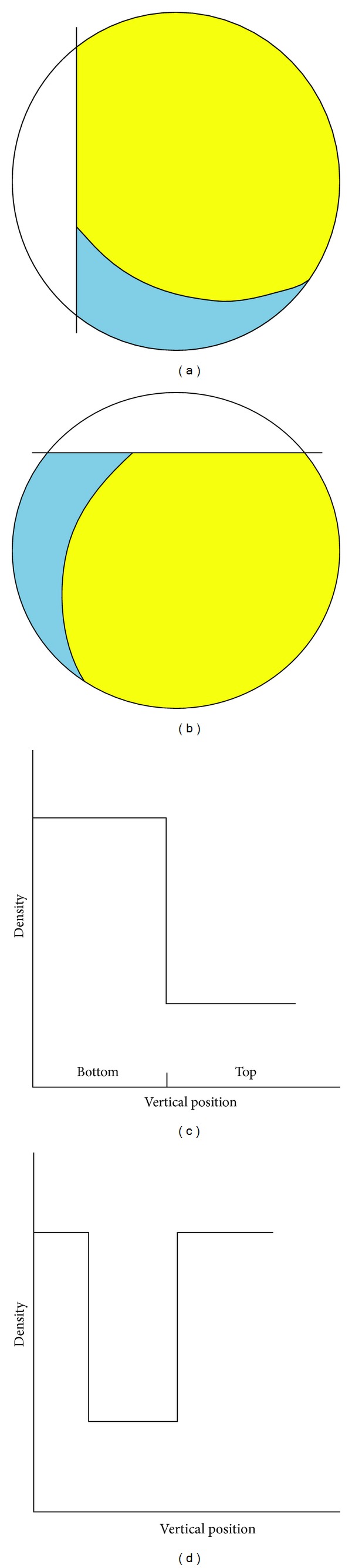
Schematic representation of the eye after vitrectomy and gravitational instability induced by the movement of the head. The blue and yellow colors indicate water silicone, respectively. In the graphs below each “eye” represents density profiles measured along a vertical axis.

Thermodynamic results suggest that spontaneous emulsification process cannot be attributed to the interfacial tension. As a consequence emulsions formation is controlled almost entirely by hydrodynamics factors. Following a vitrectomy intervention only 80–90% of the vitreous is removed; therefore the injection of silicone will form a two-phase system. Such a system is initially gravitationally stable; that is, the heavier fluid is below the lighter one; however, the eye and head movements can reverse this situation. Since head movements are much faster than the readaptation of the fluids in the eye, a gravitational instability is generated (i.e., the heavy fluid may temporarily top the light one) triggering a finger-like convective motion [25, 26]. Such instability receives its energy from the work done by the normal component of gravity at the interface. The major part of this energy is used to overcome the restoring effect of the interfacial tension and dissipation; the remainder is converted into kinetic energy. For the sake of simplicity, let us first consider an extreme type of stratification, namely, a two-layer system (Figure 2), in which initially a lighter layer of silicon floats over another heavier water layer (Figure 2(a)). The density profile (Figure 2(c)) along a vertical axis crossing the interface (for the sake of simplicity is assumed to be flat) is always stable. A head movement leads the eye in the instable configuration (Figure 2(b)). The corresponding density profile (Figure 2(d)) exhibits a* fall *in the point where silicone is sandwiched between two layers of water. This fall becomes an* attractor* for the water (heavy liquid) that acquires greater kinetic energy and the surface area of water/silicone increases. Of course, the fall does not instantly produce the emulsion, which depends on the time of permanence in that configuration, on the density and viscosity differences between the phases, and on the interfacial tension.

Formation of emulsions may also be favored by a continuous circulation of aqueous humor produced by the ciliary body and drained through the usual routes of outflow. Aqueous humor flow is tangent to the interface water-tamponade and this may induce the deformation of the interface. Indeed, physical principles tell us that gravity waves can propagate on the interface separating these two layers, but if the layers flow at different rate (i.e., when a shear is present), these waves may grow in time and lead to overturning in the vicinity of the interface. These breaking internal waves generate mixing over a height a little shorter than their wavelength (*Kelvin-Helmhotz instability*) [27]. Viscosity-induced instability finds its origin in a viscosity difference between the fluids, creating a jump in the basic-state velocity profile at the interface. Gravity-induced instability originates at the interface and receives its energy from the work done by the component of gravity in the direction of the primary flow in contrast to Rayleigh-Taylor instability, which is driven by the component of gravity perpendicular to the interface. It should be clear, however, that the effect of viscosity jump and effect of density are* coupled*; that is, it is not possible to separate viscosity-induced from gravity-induced instability, in as much as they are different manifestations of the same physical phenomenon. In which way can gravitational instability trigger the process of emulsification? A possible explanation is the formation of the interfacial tension gradients which result in* Marangoni effect* [28]. If the interface is locally curved, the concave side of the phase provides the surfactant and the curved part will have a higher interfacial tension, since it receives the smallest quantity of surfactant molecules per unit surface area. Hence interfacial transport of surfactant and a flow of liquid dragged towards the point of the strongest curvature will occur leading to an instable situation. A special discussion deserves temperature. Indeed, in recent years it was shown that, during the vitrectomy surgery, the temperature varies by several degrees [29, 30]. This may produce a gradient of density and viscosity to trigger a gravitational instability,* Benard-Rayleig instability* [31]. Since the convection flow has to vanish at the interface between adjacent* rolls*, the emulsion at the interface, and therefore the interface itself, rises at the coalescence velocity. If the temperature gradient is too large, the hydrodynamic torque exerted by counterpropagating flows meeting at an interface exceeds the gravitational restoring torque and destabilizes the interface.

## 7. Looking the Future

Intravitreous injection of silicone is considered useful for desperate cases of retinal detachment in which more convectional procedures have failed. Vitrectomy with silicone oil removal is also a preferred choice when dealing with retinal detachments occurring because of penetrating traumas especially for breaks which are too posterior to be adequately covered by an explant. The removal of silicon is performed when the silicone oil has completed its function to reattach the retina, usually after 3–6 months. However, there are many elements that speed up or delay the silicone removal (eye pressure, cataracts, vitreoretinal proliferation, and emulsification) [32]. These issues associated with silicone have stimulated the development of new blends using combinations of silicone oil and other liquids. Heidenkummer et al. investigated the emulsification rate of eight silicone oils with specific physicochemical features [33]. They observed that high contents of hydroxyl end groups enhanced silicone-oil emulsification to a greater extent than did phenyl side groups [33]. Their conclusion is in agreement with our theoretical remarks. Indeed, hydroxyl end groups decrease the silicone oil hydrophobicity, then bind more strongly water molecules reducing the interfacial turbulence. In short, it reduced the starting rate of instability. More recently, Caramoy et al. [34] studied the viscoelastic behavior of silicone oils and concluded that blends of silicone oil and high molecular mass silicone oil can be used as endotamponade in vitreoretinal surgery. These novel materials have the same viscosity of silicone oils but a lower tendency to emulsification. Once again, the cause is not attributable to the interfacial tension but the local elasticity. This in turn is due to dilatational elasticity, namely, a hydrodynamic property. In recent years, research is moving towards blends of high molecular weight silicones. Combining two liquids, the solution takes advantage of the high density of silicone blend but it can be challenging to remove. Currently, it is being removed using strong active aspiration through a long 18-gauge needle just above the optic disc, which increases the risk of iatrogenic damage to the optic nerve. Understanding what are the conditions that stabilize the system means to find the conditions to avoid the formation of foam. In the future, we will try to use a biodegradable antifoam to reduce the formation of emulsions without losing the advantage of a low molecular mass silicone oil.

## 8. Conclusion

We propose that gravitational instabilities play the main role for the formation of emulsions in vitrectomized eyes filled with liquid tamponades. The instability is induced by tangential disturbances originated at the interface and is driven by the rate at which work is done by the velocity and stress disturbances in the direction of primary flow. Theoretical remarks performed herein have shown that the spontaneous emulsification of silicone oil is not due to lowering of the surface tension but due to a hydrodynamic instability. Our theoretical results seem in line with the experimental evidences; furthermore they indicate a future research line for the improvement of endotamponades. Indeed, the use of biodegradable antifoam may avoid the formation of bubbles and delay the formation of emulsions.
